# Exploration of ovine milk whey proteome during postnatal development using an iTRAQ approach

**DOI:** 10.7717/peerj.10105

**Published:** 2020-10-08

**Authors:** Xueying Zhang, Fadi Li, Fang Qin, Wanhong Li, Xiangpeng Yue

**Affiliations:** 1 State Key Laboratory of Grassland Agro-ecosystems; Key Laboratory of Grassland Livestock Industry Innovation, Ministry of Agriculture and Rural Affairs; Engineering Research Center of Grassland Industry, Ministry of Education; College of Pastoral Agriculture Science and Technology, Lanzhou University, Lanzhou, Gansu, China; 2 Engineering Laboratory of Sheep Breeding and Reproduction Biotechnology in Gansu Province, Minqin, Gansu, China; 3 School of Pharmacy, Lanhzou University, Lanzhou, Gansu, China

**Keywords:** Hu sheep, iTRAQ, LC-MS/MS, Proteomics, Whey protein

## Abstract

**Background:**

Ovine milk is a rich source of bioactive proteins that supports the early growth and development of the newborn lambs. A large number of researches had targeted to the identification of ovine milk fat globule membrane proteins (MFGMPs), caseins (CNs), mastitis milk proteins in past years, but the dynamic change tendency of milk whey proteins during postnatal development has received limited attention. This research aimed to investigate the dynamic changes of ovine milk whey proteins after delivery, and explore the functions of whey proteins on early development of the newborns.

**Methods:**

In this research, Hu sheep milk samples were collected from six individuals by manual milking manner, at 0 d, 3 d, 7 d, 14 d, 28 d and 56 d after delivery, respectively. The milk whey proteins were identified and quantified by the isobaric tag for relative and absolute quantification (iTRAQ) coupled with liquid chromatography (LC)-electrospray ionization (ESI) tandem MS (MS/MS) methods. In addition, biological functions of differentially expressed proteins (DEPs) were annotated by Gene Ontology (GO) annotation and Kyoto Encyclopedia of Genes and Genomes (KEGG) pathway enrichment analysis.

**Results:**

A total of 310 proteins were identified , of which 121 were differentially expressed. In detail, 30 (10 up-regulated and 20 down-regulated), 22 (11 up-regulated and 11 down-regulated), 11 (four up-regulated and seven down-regulated), 11 (eight up-regulated and three down-regulated), 10 (six up-regulated and four down-regulated) DEPs were identified in 3 d vs. 0 d, 7 d vs. 3 d, 14 d vs. 7 d, 28 d vs. 14 d, 56 d vs. 28 d comparison groups, respectively. The GO annotation analysis revealed that biological process principally involved metabolic and biological regulation, the major cellular location were organelle, cell and extracellular region, and the mainly molecular function were binding and catalytic activity. Circadian rhythm, fatty acid biosynthesis and African trypanosomiasis were enriched by KEGG annotation analysis.

**Conclusion:**

The study reveals a comprehensive understanding of Hu sheep milk proteome, suggesting whey proteins change dramatically in early development of newborn lambs, which provide a potential guidance for early weaning of lambs.

## Introduction

Milk as a complex body fluid synthesized by mammals, contains a large number of secreted proteins that contribute to the growth, development and immune system of neonates (*Roncada et al., 2012*; Hernandez-Castellano et al., 2015b; Li et al., 2019). Sheep milk contains a wide range of proteins, including the high-abundance proteins α_s1_-casein (α_s1_-CN), α_s2_-casein (α_s2_-CN), β-casein (β-CN), κ-casein (κ-CN), β-lactoglobulin (β-Lg) and α-lactalbumin (α-Lac; Gidlund et al., 2015) and low-abundance proteins such as immunoglobulin, lactoferrin, hormones and enzymes (Le et al., 2011). The proteins in mammalian milk can be mainly divided into three classes: caseins (CNs), whey proteins and milk fat globule membrane proteins (MFGMPs). Among these classes, CNs are primary proteins that present in micellar form, whereas whey fractions occur in soluble form (*Roncada et al., 2012*). MFGMPs are mainly present in the cream portion of milk, which is composed by proteins and lipids at a 1:1 weight ratio (*Yang et al., 2016; Li et al., 2020*). These three different protein fractions can be separated by centrifugation for proteomics analysis of raw milk (*Roncada et al., 2012*).

Among the three protein fractions described above, whey accounts for approximately 13∼20% of the total proteins in sheep milk and is generally regarded as having measurable effects on health outcomes such as immunomodulatory, antimicrobial properties, acute inflammatory response, complement activation and innate immune response (*Hernández-Ledesma, Ramos & Gómez-Ruiz, 2011*; Ha et al., 2015). In addition, the natural digestion of sheep whey proteins in the gastrointestinal tract can generate peptides with a variety of bioactivities, for instance, antihypertensive, opioid, antibacterial, antioxidant, and immunomodulatory activities (*Recio & Visser, 2000*; Tina et al., 2010). The proteome of cow and sheep whey has attracted extensive research. To date, up to 606 proteins have been identified in sheep whey, while 783 proteins have been found in cow whey (*D’Alessandro, Zolla & Scaloni, 2011; Le et al., 2011; Nissen et al., 2013; Reinhardt et al., 2013; Ha et al., 2015*). A comparative analysis between the two whey proteomes revealed only 233 common proteins, most of which were associated with immunity (*Ha et al., 2015*). Gene Ontology (GO) analysis of whey proteins unique to the cow revealed significant involvement in cellular development and metabolism, while proteins unique to sheep were found to function in cellular establishment, signaling, protein maturation, and inflammatory and other immune responses (*Ha et al., 2015*). Previous proteomic studies on sheep milk have mainly focused on the characterization of the sheep colostrum proteome, MFGMPs, skimmed milk and whey proteins at specific time points (*Pisanu et al., 2012; Hernandez-Castellano et al., 2015a*) and the proteomic analysis of sheep milk to detect mastitis (*Chiaradia et al., 2013*). The available differential proteomic studies of milk proteins at different lactation stages have mainly been conducted in bovines (*Murgiano et al., 2009; Zhang et al., 2015; Tacoma et al., 2016*), while very few such studies have been reported for sheep milk. The changes of protein constituents in the milk whey proteome during the transition from colostrum to mature milk are therefore important for a more detailed understanding of the biological significance of milk whey and can supply a reference for developing lamb weaning strategies and milk replacer formula.

In China, milk production is dominated by cows and dairy goats, while sheep milk is mostly exclusively consumed by newborn lambs and is not used for human consumption. Local sheep breeds in China are usually highly fecund. Hu sheep are excellent representatives of Chinese sheep and are the most widely distributed sheep breed in China. In most cases, ewes of this breed can give birth to 2∼4 lambs per parity and can produce three lambs biennially (Yue, 1996). In addition, Hu sheep can produce 120–150 kg milk in 120–150 days of lactation, which is essential for a high survival rate and fast growth rate of Hu lambs after birth (*Yue, 1996*). Proteomics have been used widely and effectively to analyze milk-derived proteins, and proteomic technologies have greatly advanced our in-depth knowledge of milk proteins (*O’Donnell et al., 2004*). In this study, the isobaric tags for relative and absolute quantification (iTRAQ) method was used to explore whey proteome changes in Hu sheep at the six time points of 0, 3, 7, 14, 28 and 56 d after delivery. The method of hierarchical clustering was used to analyze the significantly differentially expressed proteins in the different lactation stages. GO annotation and Kyoto Encyclopedia of Genes and Genomes (KEGG) analysis were used to analyze the biological function of differentially expressed proteins (DEPs). The objective of this research was to detect the protein profile of sheep milk during early lactation (within 56 d after delivery) and to provide potential guidance for the early weaning of Hu lambs.

## Materials & Methods

### Ethics statement

The experimental procedures were approved by the ethics committee of College of Pastoral Agriculture Science and Technology of Lanzhou University. All efforts were taken to minimize animal suffering.

### Sample collection and whey protein preparing

Six multiparous healthy Hu sheep (Zhongtian sheep Ltd., Jinchang, Gansu Province, China) were selected after brucellosis and mastitis testing. Mastitis was identified on the basis of clinical signs (heat, pain, redness and swelling of the udder, or clots in the milk), as well as the corresponding somatic cell counting parameters. All the ewes given the second birth and with the litter size of two, and were reared at the same condition and fed by the same feed during the milking period in the present study. The fresh milk samples from the whole available milk were individually collected by manual milking manner, at 8:00–9:00 on 3 d, 7 d, 14 d, 28 d, 56 d and 4∼5 h (0 d) after delivery. Fresh milk was immediately frozen at −20 °C until whey preparation. Whole-milk samples were defatted by centrifugation at 3,000× g and 4 °C for 15 min (Biofuge Stratos, Heraeus). The precipitated casein was further removed by ultracentrifugation at 100,000× g and 4 °C for 60 min (CS120GXL, Hitachi) to obtain the milk whey fraction (*Yang et al., 2013*). Thereafter, STD buffer (4% SDS, 1 mM DTT, 150 mM Tris-HCl pH 8.0) were added into the collected supernatant, and the mixed solution were heated at 95 °C for 5 min. The proteins concentration was determined by bicinchoninic acid (BCA) Protein Assay Kit (PC0020, Solarbio Ltd., Beijing) according to manufacturer’s instructions with bovine serum albumin (BSA) as a standard for calibration curve.

### Protein digestion and iTRAQ labeling

The filter-aided sample preparation (FASP) procedure was used to digest the whey protein (*Wisniewski et al., 2009*). Total-proteins (300 µg) for each sample were mixed with UA buffer (200 µL, 8 M Urea, 150 mM Tris-HCl pH 8.0), and loaded on an ultrafiltration filter (Pall units, 10 kD). The detergent, DTT and other low-molecular-weight components were removed by repeated centrifugation at 14,000× g for 15 min. Then, 100 µL of iodoacetamide solution (50 mM iodoacetamide in UA buffer) were added and the samples were shocked at 600 rpm for 1 min, followed by incubation in the dark for 30 min and centrifuged at 14,000× g for 10 min. Using 100 µL UA buffer and 100 µL Dissolution buffer (50 mM triethylammonium bicarbonate at pH 8.5) to wash the filters by centrifuged at 14,000× g for 10 min, and every wash step was repeated twice. Finally, 2 µg Trypsin (Promega, Southampton, UK) were incorporated into 40 µL Dissolution buffer and the solution was used to digest the protein suspensions at 37 °C for 16–18 h. The filtrate was collected after centrifugation at 14,000× g for 10 min. Using UV light spectral density at 280 nm to estimate the concentration of the resulting peptides.

Subsequently, the 8-plex iTRAQ reagent kit (Applied Biosystems, Forster City, CA) was used to label the resulting peptide mixture. The labeling procedure was carried out according to the manufacturer’s instructions. The protein samples were labeled as (Sample 0 d)-113, (Sample-3 d)-114, (Sample-7 d)-115, (Sample-14 d)-116, (Sample-28 d)-117, (Sample-56 d)-118, and the labeling solution reaction was incubated at room temperature for 60 min.

### Peptide fractionation with strong cation exchange chromatography

All of the labeled peptides were mixed and then fractionated by strong cation exchange (SCX) chromatography using AKTA Purifier 100 system (GE Healthcare). The used column was Polysulfoethyl 4. 6 ×100 mm column (5 µm, 200 Å) (PolyLCInc, Maryland, USA), and solvent A was 10 mM KH_2_PO_4_ pH 3.0 in 25% of ACN, solvent B was 500 mM KCl, 10 mM KH_2_PO_4_ pH 3.0 in 25% of ACN. The solvents were applied using the time gradient from 0–100% solvent B and followed by 10 min at 0%. A total of 36 fractions were collected and finally combined into 15 pools. C _18_Cartridges (66872-U, Sigma) was used to desalt the freeze-dried samples and all samples were stored at −80 °C until LC-MS/MS analysis.

### Liquid chromatography (LC)-electrospray ionization (ESI) tandem MS (MS/MS) analysis by Q Exactive

The liquid chromatography was carried out using a Easy nano-LC system (Proxeon Biosystems, now Thermo Fisher Scientific), which was coupled with a Q Exactive mass spectrometer. Chosen 0.1% Formic acid as buffer A, and 80% acetonitrile with 0.1% Formic acid as buffer B. The chromatographic column was balanced by 95% buffer A. 10 µL of peptide mixture was loaded onto the Thermo scientific EASY column (two cm ×100 µm 5 µm-C18), and then separated by analytical column (75 µ×100 mm 3 µm-C18) at a flow rate of 250 nL/min. Peptides were eluted using a 60 min gradient with 0% B to 40% B over 55 min, 40% B to 100% B for 3 min, and held at 100% B for 2 min.

Mass spectrometry analyses were performed on Q-Exactive (Thermo Finnigan, San Jose, CA). The data was acquired in the positive ion mode, and the total analytical time was 60 min. Mass range was 300–1,800 m/z with the resolving power of 70,000 at m/z 200 for the MS scan, and mass range was 200–2,000 with the resolution of 17,500 at m/z 200 for the MS/MS scan. Dynamic exclusion duration was 60 s. The maximum ion injection time for the survey scan was 10 ms, and 45 ms in MS/MS scan. Top 10 most abundant ion maps were collected after scan. Normalized collision energy was 30 eV and the underfill ratio was defined as 0.1%. The instrument was run with peptide recognition mode enabled.

### Sequence database searching and data analysis

Raw data files were submitted to MASCOT engine (Matrix Science, London, UK; version 2.2) through Proteome Discoverer 1.4 (Thermo Electron, San Jose, CA.). The MS/MS spectra were searched against UniProt *ovis aries* (27065 sequences, download at 09, 10, 2015) and UniProt *cetartiodactyla* (712254 sequences, download at 15, 10, 2015). For protein identification, the following options were used: Peptide mass tolerance=20 ppm, MS/MS tolerance=0.1 Da, Enzyme=Trypsin, Missed cleavage=2, Fixed modification: Carbamidomethyl (C), iTRAQ 8 plex (K), iTRAQ 8 plex (N-term), Variable modification: Oxidation (M), FDR ≤ 0.01.

### Bioinformatic analysis

The selected differentially expressed proteins (DEPs) were in batches matched in UniProtKB database (Release 2019_10) to retrieve the sequence data in FASTA format. To find homologue sequences, the retrieved sequences were locally searched against SwissProt database (mammal) using the NCBI BLAST+ client software (ncbi-blast-2.2.28+-win32.exe). GO mapping and annotation was conducted by Blast2GO (Version 2.7.2; (Gotz et al., 2008; Ashburner et al., 2000) under the configuration: an *E*-value filter of 1*e*-6, default gradual EC weights, a GO weight of 5, and an annotation cutoff of 55. The KOs of the DEPs were blasted against KEGG GENES (mammal), and then the pathway analysis was conducted by mapping the KOs in KEGG (*Kanehisa et al., 2012*).

## Results

### Identification of differential proteins in the whey fractions of Hu sheep after delivery

The iTRAQ-LC-MS/MS proteomics analysis conducted in this study resulted in the identification of 1,068 unique peptides and 310 proteins in Hu sheep milk whey at the six time points of 0, 3, 7, 14, 28 and 56 d after delivery. Among these proteins, 111 were directly matched to the sheep proteomics database, and 199 proteins were matched to the vertebrate proteomics database (Table S1). The pairwise comparison of the six time points identified 121 DEPs (Table S2). The analysis of whey protein expression levels between adjacent time points revealed 30 (10 up-regulated and 20 down-regulated), 22 (11 up-regulated and 11 down-regulated), 11 (four up-regulated and seven down-regulated), 11 (eight up-regulated and three down-regulated), 10 (six up-regulated and four down-regulated) DEPs in the 3 d vs. 0 d, 7 d vs. 3 d, 14 d vs. 7 d, 28 d vs. 14 d, and 56 d vs. 28 d comparison groups, respectively (Tables 1–5).

**Table 1:** The differentially expressed proteins between 3 d and 0 d.

NO.	Accession no.	Protein name	Fold change^a^	*P* value
1	G3LUQ4	alpha s1 casein	3.97	3.33E−04
2	P04654	alpha-s2-casein precursor	3.47	1.19E−03
3	W5Q6B8	low quality protein: type ii cytoskeletal 6a isoform x1	3.30	1.87E−03
4	W5PF70	low quality protein: biorientation of chromosomes in cell division protein 1-like 1 isoform x1	3.19	2.48E−03
5	W5NQP5	superoxide dismutase [Cu-Zn]	2.72	9.09E−03
6	W5NXZ8	g protein- regulated inducer of neurite outgrowth 1	2.28	3.12E−02
7	E7BQS5	alpha-s2-casein variant	2.28	3.17E−02
8	W5NUD7	collectin43 isoform x1	2.15	4.62E−02
9	W5QB61	peptidyl-prolyl cis-trans isomerase	2.13	4.88E−02
10	W5QDQ1	predicted: uncharacterized protein C1 or f94 homolog	2.03	6.43E−02
11	W5PHP6	60s ribosomal protein l27	0.48	8.75E−02
12	P12303	transthyretin	0.47	7.62E−02
13	W5P0V6	saccharopine dehydrogenase-like oxidoreductase	0.47	7.62E−02
14	W5QHZ8	immunoglobulin kappa-4 light chain variable region	0.41	3.83E−02
15	W5NTD9	chitinase-3-like protein 1	0.41	3.78E−02
16	W5QAL0	glucosidase 2 subunit beta x3	0.39	2.82E−02
17	W5P673	pyruvate dehydrogenase phosphatase regulatory mitochondrial	0.38	2.41E−02
18	W5PK06	low affinity immunoglobulin gamma fc region receptor ii-like isoform x3	0.37	2.08E−02
19	W5PSP9	immunoglobulin lambda-2c light chain variable region	0.36	1.79E−02
20	W5PH95	immunoglobulin heavy chain constant region of tetrameric 1a membrane form	0.36	1.76E−02
21	W5Q3H4	ribosomal protein s2	0.36	1.70E−02
22	W5NV14	immunoglobulin v lambda chain	0.36	1.61E−02
23	W5PSQ7	immunoglobulin lambda light chain f7-299	0.34	1.12E−02
24	W5Q524	poly polymerase alpha isoform x1	0.33	1.06E−02
25	B3F206	cryptochrome 1	0.33	9.35E−03
26	W5PFM6	iq domain-containing protein d	0.31	6.61E−03
27	W5PGT9	immunoglobulin epsilon- partial	0.30	4.64E−03
28	W5NUN9	mortality factor 4 like 1	0.27	2.07E−03
29	W5QHZ5	Ig k protein	0.22	4.28E−04
30	W5PXV3	connective tissue growth factor	0.16	2.45E−05

**Notes.**

a Relative abundance of whey proteins in 3 d versus 0 d.

**Table 2:** The differentially expressed proteins between 7 d and 3 d.

NO.	Accession no.	Protein name	Fold change^a^	*P* value
1	W5NTK7	transmembrane protein c15orf27 homolog isoform x1	6.00	1.03E−10
2	W5NTD9	chitinase-3-like protein 1	2.92	9.39E−05
3	W5NVL1	lysosomal-associated transmembrane protein 5	2.83	1.44E−04
4	C7DLN1	fatty acid synthase (fragment)	2.55	5.95E−04
5	P68214	fibrinogen alpha chain (fragment)	2.44	1.08E−03
6	W5PZI1	clusterin	2.22	3.26E−03
7	W5QFH5	ras-related protein rab-1a	2.17	4.26E−03
8	W5PHP6	60s ribosomal protein l27	2.03	8.70E−03
9	D3G9G3	lactoferrin precursor	2.02	9.25E−03
10	P68116	fibrinogen beta chain (fragment)	2.01	9.79E−03
11	W5PN88	elongation factor 2	2.01	9.84E−03
12	B5B304	complement factor H (fragment)	0.47	3.64E−03
13	C6ZP47	I alpha globin	0.41	5.88E−04
14	W5PF70	low quality protein: biorientation of chromosomes in cell division protein 1-like 1 isoform x1	0.39	2.76E−04
15	W5Q4D0	cyclin-g-associated kinase x6	0.38	1.69E−04
16	W5PXV3	connective tissue growth factor	0.37	1.25E−04
17	H9A6H7	myostatin variant A	0.35	5.46E−05
18	W5PJG0	serum amyloid A protein	0.32	1.07E−05
19	G3LUQ4	alpha s1 casein	0.31	6.31E−06
20	P02075	hemoglobin subunit beta	0.27	4.49E−07
21	W5NSJ5	serine threonine-protein kinase osr1 isoform x1	0.26	9.61E−08
22	W5QA36	lactase-like protein	0.19	6.63E−11

**Notes.**

a Relative abundance of whey proteins in 7 d versus 3 d.

**Table 3:** The differentially expressed proteins between 14 d and 7 d.

NO.	Accession no.	Protein name	Fold change^a^	*P* value
1	W5PDM2	fibroblast growth factor-binding protein 1	4.74	1.19E−07
2	W5PF70	low quality protein: biorientation of chromosomes in cell division protein 1-like 1 isoform x1	3.24	7.52E−05
3	W5P0V6	saccharopine dehydrogenase-like oxidoreductase	2.53	1.99E−03
4	D2DRB7	alpha-s1-casein variant	2.26	7.27E−03
5	W5NSJ5	serine threonine-protein kinase osr1 isoform x1	0.50	4.09E−02
6	W5NTK7	transmembrane protein c15orf27 homolog isoform x1	0.46	2.33E−02
7	W5PZI1	clusterin	0.42	1.14E−02
8	W5PYA9	upstream-binding factor 1-like protein 1	0.41	1.08E−02
9	E7BQS3	alpha-s2-casein precursor	0.40	7.86E−03
10	D3G9G3	lactoferrin precursor	0.37	4.47E−03
11	W5NTD9	chitinase-3-like protein 1	0.23	3.16E−05

**Notes.**

a Relative abundance of whey proteins in 14 d versus 7 d.

**Table 4:** The differentially expressed proteins between 28 d and 14 d.

NO.	Accession no.	Protein name	Fold change^a^	*P* value
1	G3LUQ4	alpha s1 casein	10.78	3.53E−22
2	Q9XSC0	beta-lactoglobulin C	3.08	4.27E−06
3	E7BQS5	alpha-s2-casein variant	2.82	2.24E−05
4	W5P559	odorant-binding protein 2b	2.65	6.51E−05
5	W5NXZ8	g protein-regulated inducer of neurite outgrowth 1	2.62	8.05E−05
6	W5PZS7	alpha-1-antiproteinase isoform x1	2.39	3.43E−04
7	W5NSJ5	serine threonine-protein kinase osr1 isoform x1	2.36	4.39E−04
8	W5Q629	protein ddi1 homolog 1	2.05	3.29E−03
9	W5P7S6	alpha-1-acid glycoprotein	0.48	9.41E−03
10	W5PZM9	annexin	0.42	1.92E−03
11	W5PDM2	fibroblast growth factor-binding protein 1	0.28	5.26E−06

**Notes.**

a Relative abundance of whey proteins in 28 d versus 14 d.

**Table 5:** The differentially expressed proteins between 56 d and 28 d.

NO.	Accession no.	Protein name	Fold change^a^	*P* value
1	P04653	alpha-s1-casein variant	10.72	1.40E−36
2	W5P3X8	kinesin-like protein	3.10	2.55E−09
3	W5Q524	poly polymerase alpha isoform x1	2.70	1.72E−07
4	W5Q293	dynein heavy chain axonemal isoform x2	2.51	1.30E−06
5	W5PDJ7	60s ribosomal protein l5	2.44	2.82E−06
6	W5PJA0	protein isoform x4	2.39	5.03E−06
7	W5P559	odorant-binding protein 2b	0.43	1.97E−05
8	Q9XSC0	beta-lactoglobulin C (fragment)	0.36	3.86E−07
9	W5PZS7	alpha-1-antiproteinase isoform x1	0.11	2.56E−28
10	G3LUQ4	alpha s1 casein	0.10	6.09E−31

**Notes.**

a Relative abundance of whey proteins in 56 d versus 28 d.

### Gene ontology analysis of the DEPs

To explore the regulatory mechanism of milk proteins and verify the potential molecular associations among DEPs, we performed GO functional annotation analysis of these proteins according to molecular function, cellular location and biological pathways.

**Figure 1:**
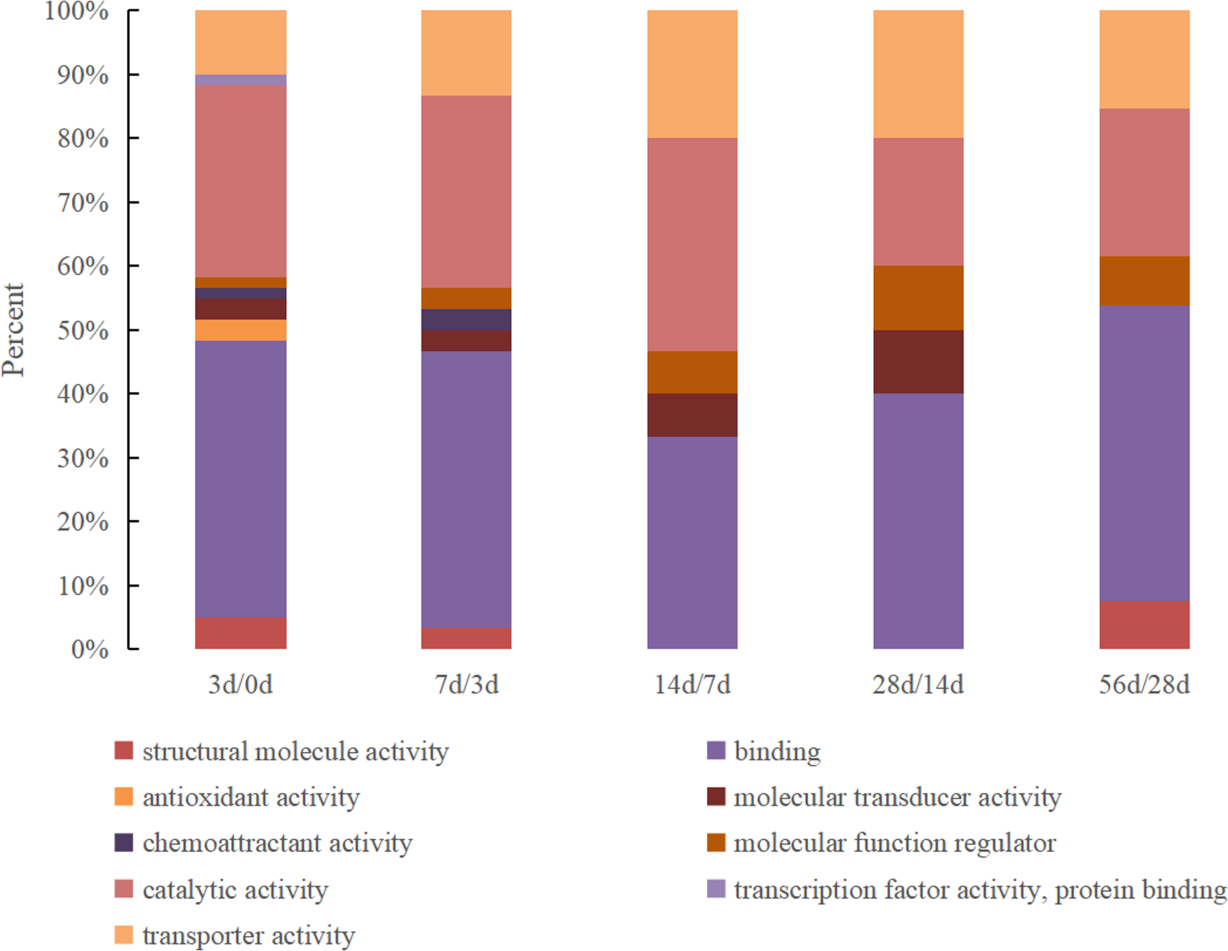
The GO molecular function classification of DEPs in Hu sheep at different lactation stages. (A) 3 d vs. 0 d; (B) 7 d vs. 3 d; (C) 14 d vs. 7 d; (D) 28 d vs. 14 d; (E) 56 d vs. 28 d.

The DEPs were classified according to molecular functions between the milk samples from every two adjacent time points, and the results are shown in Fig. 1. The DEPs identified between 0 d and 3 d presented the most comprehensive molecular functions and were enriched in nine specific functional terms, with the majority being related to binding activity and catalytic activity (accounting for 43% and 30%, respectively), followed by transporter activity, structural molecular activity, antioxidant activity and molecular transducer activity. Other proteins were associated with molecular function regulator, chemoattractant activity and transcription factor activity. The number of molecular function categories of the DEPs in the 3 d vs. 7 d group decreased to 7 that antioxidant activity and transcription factor activity were lost. Furthermore, the function of structural molecular activity was lost in the 7 d vs. 14 d and 14 d vs. 28 d groups but existed in the 28 d vs. 56 d group. Chemoattractant activity only existed in the 0 d vs. 3 d and 3 d vs. 7 d groups.

**Figure 2:**
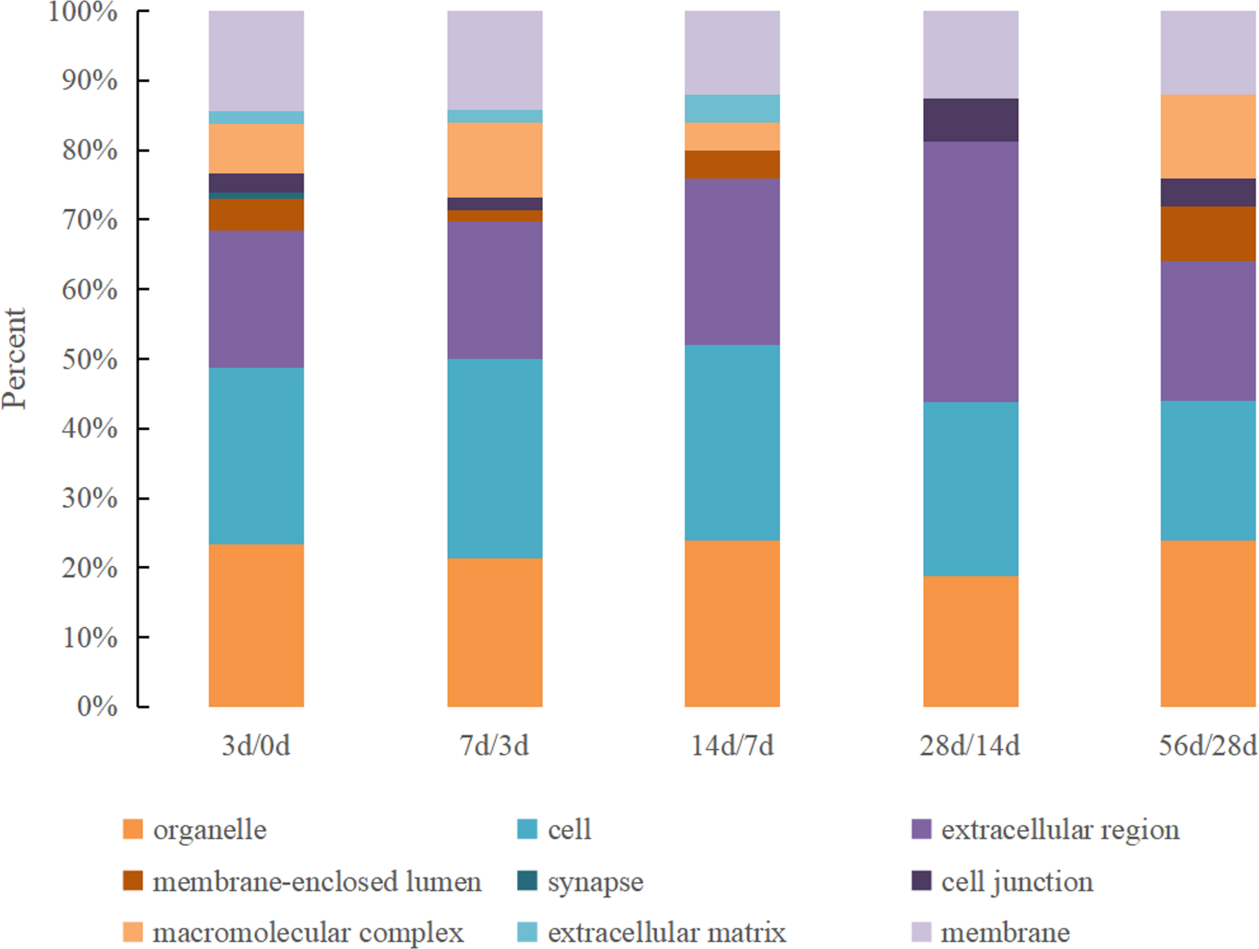
The GO cellular location classification of DEPs in Hu sheep at different lactation stages. (A) 3 d vs. 0 d; (B) 7 d vs. 3 d; (C) 14 d vs. 7 d; (D) 28 d vs. 14 d; (E): 56 d vs. 28 d.

The GO cellular location classifications of the DEPs identified in Hu sheep at different lactation stages were mainly involved in organelle, cell, extracellular region, membrane-enclosed lumen, synapse, cell junction, macromolecular complex, the extracellular matrix and membrane (Fig. 2). All nine cellular location categories were included in the 0 d vs. 3 d group, and the kinds decreased gradually in other comparison groups. The most prevalent cellular locations were organelle, cell and extracellular region, which together accounted for over 60% in all groups.

**Figure 3:**
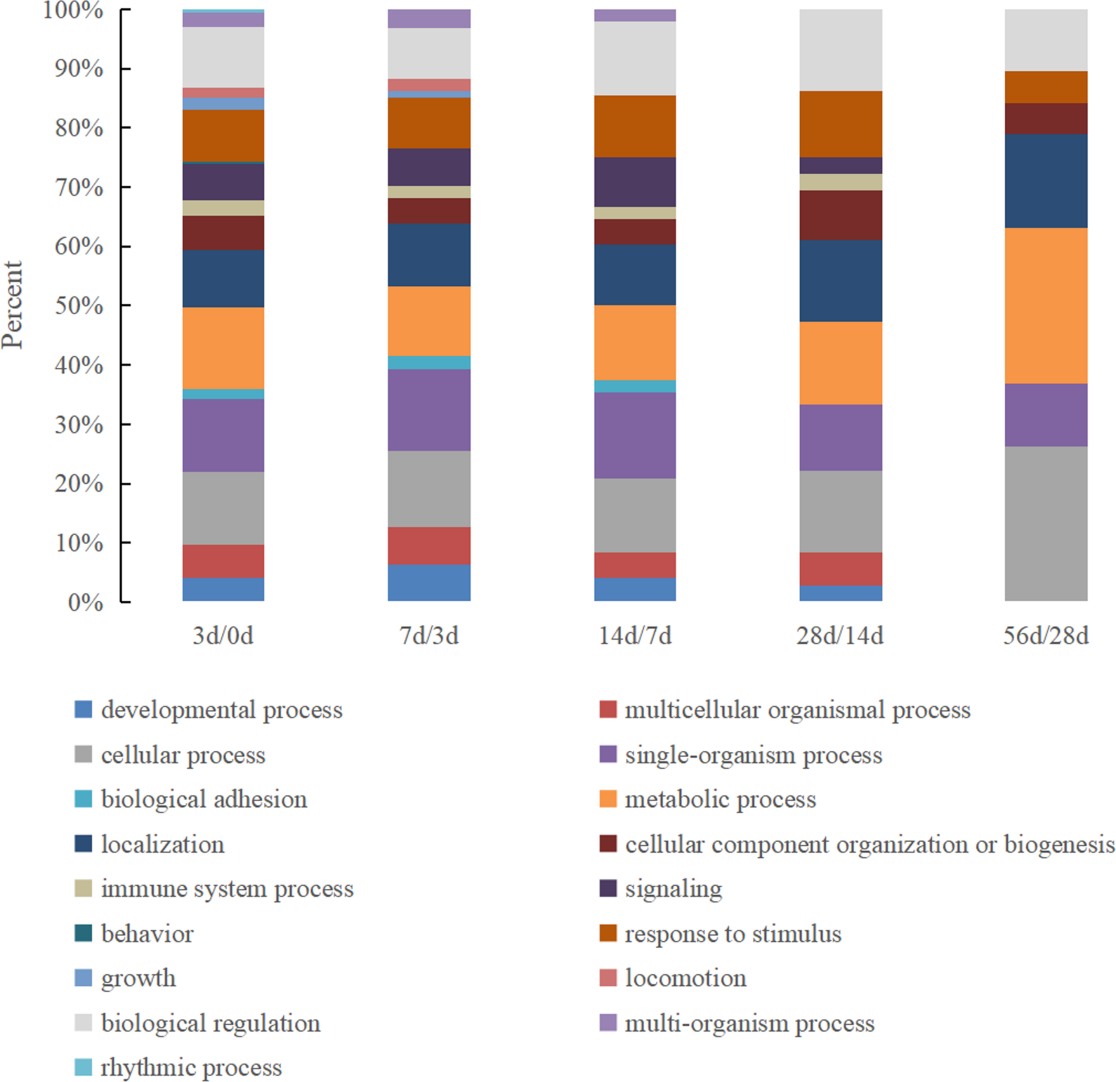
The GO biological process classification of DEPs in Hu sheep at different lactation stages. (A) 3 d vs. 0 d; (B) 7 d vs. 3 d; (C) 14 d vs. 7 d; (D) 28 d vs. 14 d; (E) 56 d vs. 28 d.

There were 17 biological process categories in which the identified DEPs were involved (Fig. 3). The proteins in the 0 d vs. 3 d group exhibited the most complete classifications, and six biological processes exhibited a major change between the remaining comparison groups: behavior, rhythmic process, growth, biological adhesion, multi-organism process and locomotion.

### KEGG pathway analysis of the identified proteins

KEGG pathway analysis was also conducted for the DEPs identified by the comparison of the milk whey proteins at adjacent time points. As shown in Table 6, the set of proteins was enriched in three pathways: circadian rhythm was enriched in the 3 d vs. 0 d group, and fatty acid biosynthesis and African trypanosomiasis were enriched in the 7 d vs. 3 d group.

### Cluster analysis of the differentially expressed whey proteins

**Figure 4:**
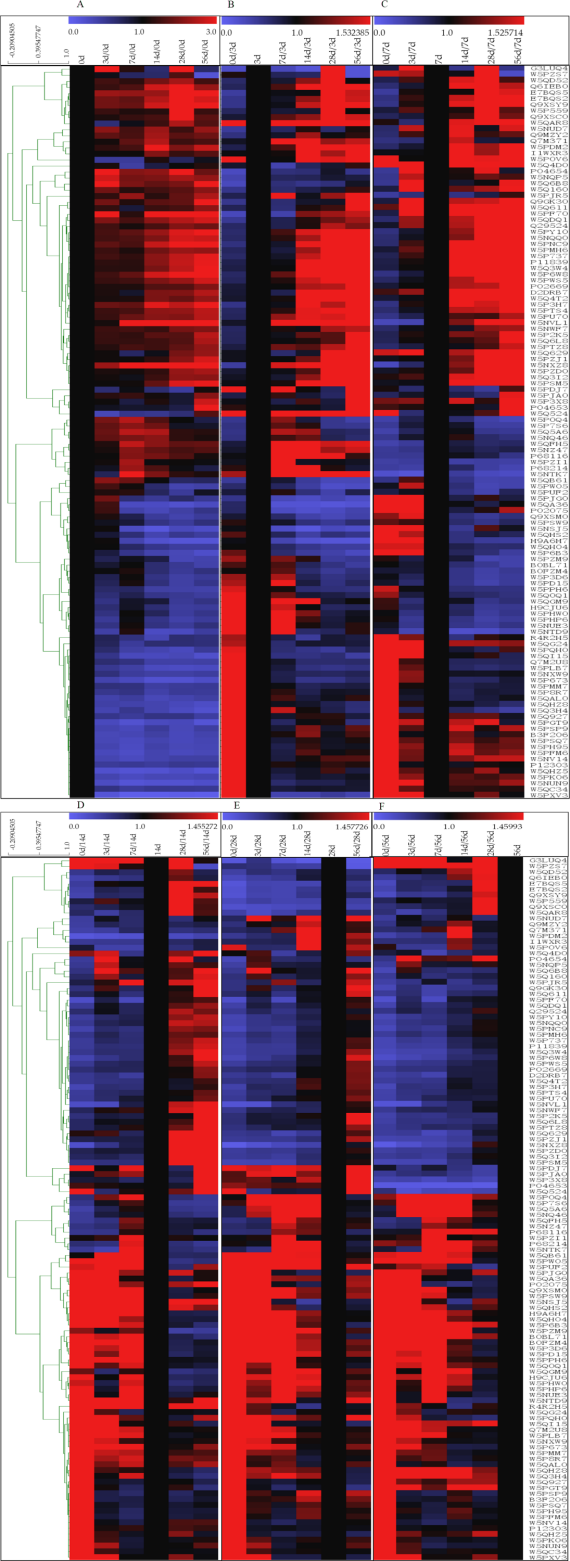
Hierarchical clustering of DEPs. (A–F) means 0, 3, 7, 14, 28 and 56 d as the reference, based on the level of protein affinity, the clustering analysis of the DEPs. The color bars from blue to red represents scale 0∼3, 0∼1.532, 0∼1.526, 0∼1.455, 0∼1.458, 0∼1.460, respectively. Blue represented decreasing protein abundance, red represented increasing protein abundance.

The 121 identified DEPs were analyzed by hierarchical clustering analysis based on their affinity (Fig. 4). The high-abundance proteins α-La and β-Lg exhibited low expression at 0 d, while both of these proteins presented their highest contents at 28 d, which were approximately 2.4 and 4.7 times higher, respectively, than that at 0 d. Expression level changes were also observed for low-abundance proteins between the 6 lactation stages, such as superoxide dismutase [Cu-Zn], airway lactoperoxidase, lipoprotein lipase, and peptidyl-prolyl cis-trans isomerase. In addition, significant differences were found for proteins that were searched according to homology. Lysosomal-associated transmembrane protein 5, G protein-regulated inducer of neurite outgrowth 1 and low-quality proteins such as biorientation of chromosomes in cell division protein 1-like 1 isoform x1 presented a low concentration in 0 d and 3 d milk whey but exhibited high expression in milk at 14 d, 28 d and 56 d after delivery. Proteins such as pyruvate dehydrogenase phosphatase regulatory mitochondrial, connective tissue growth factor, immunoglobulin epsilon-partial, Ig k protein and mortality factor 4 like 1 were almost exclusively present in milk at 0 d after delivery and absent in milk samples at other time points.

**Table 6:** Pathway categories of differentially expressed proteins.

Group	Pathway name	KO ID	Protein name
3 d vs. 0 d	Circadian rhythm	oas04710	cryptochrome 1
7 d vs. 3 d	Fatty acid biosynthesis	oas00061	fatty acid synthase (fragment)
	African trypanosomiasis	oas05143	I alpha globin

## Discussion

Of those animal milk proteome related researches, most of the studies have focused on cow and goat milk, and few studies have investigated the dynamic changes in sheep milk proteins during lactation stages. The lack of such research has led to the limitation of the sheep protein database (*Hernandez-Castellano et al., 2015a*). This study analyzed the differential expression of Hu sheep milk whey proteins at different lactation stages. In our research, 121 DEPs were identified, and functional annotation and metabolic pathway annotation were performed to illustrate the molecular functions of the sheep milk whey proteins in infant growth and development. This research enriches the sheep protein database and provides a direction for the exploitation of specific milk protein production.

With the rise of proteomics technology, many studies involving milk protein quantification and qualitative analysis have been conducted, and varieties of research methods have been derived. Researchers identified 149 whey proteins in bovine milk by combinatorial peptide ligand library analysis combined with 1D-SDS-PAGE and 2D-PAGE analyses (*D’Amato et al., 2009*). Under the ion-exchange approach, 293 unique gene products were identified in bovine colostrum and mature whey (*Le et al., 2011*). In ovine whey, Scumaci et al. (2015) used a 1D-SDS-PAGE with LC-MS/MS method and identified 343 unique proteins in Appenninic sheep. Ha et al. (2015) used the ProteoMiner Kit to analyze sheep milk whey proteins in depth, and 669 proteins were identified, representing the largest inventory of sheep whey proteins identified to date. Low-abundance proteins are a kind of active substances with low expression that participate in a variety of biological metabolic processes (*Righetti, Boschetti & Monsarrat, 2007*) but may be covered by high-abundance proteins. ProteoMiner Kit technology can be used to saturate high-abundance proteins quickly and increase the number of minor peptides and proteins that are detectable by LC MS/MS, which is specifically used to analyze low-abundance proteins (*Ha et al., 2015*). The isobaric tags for relative and absolute quantification (iTRAQ) method is a quantitative proteomics approach with a relatively high throughput that allows simultaneous identification and peptide quantification by measuring the peak intensities of reporter ions via MS/MS (*Pottiez et al., 2012*). Reinhardt et al. (2013) used iTRAQ technology to quantify protein changes between milk fractions isolated from healthy and S. aureus infected cows, and 748 whey proteins were identified. In this work, we used iTRAQ technology combined with LC-MS/MS and 310 proteins were identified in Hu sheep whey milk at different lactation stages. The number of the identified proteins varies greatly in different researches, which illustrated that milk whey proteins present significant differences between species, and the chosen method also has a great influence on protein detection. The identified proteins were further quantified under the iTRAQ approach in this study, and differentially expressed proteins were found.

The DEPs identified in our research were involved in multiple biological processes, including immunity, growth, disease, lipid metabolism, and protein metabolism. A large number of proteins were indicated to function as immune modulators, such as superoxide dismutase [Cu-Zn], collectin43 isoform x1, haptoglobin isoform x1, fibrinogen alpha/beta chain, complement factor I, complement C3, immunoglobulin, serum amyloid A protein (fragment), and mannose-binding lectin. As the major immune factors, immunoglobulins identified in this research, including immunoglobulin epsilon, immunoglobulin kappa-4 light chain variable region, immunoglobulin lambda-2c light chain variable region, immunoglobulin v lambda chain and immunoglobulin alpha heavy chain, were found to be differentially expressed at different lactation stages, and the expression levels of these proteins were significantly decreased in mature milk. Thus, colostrum is the main source of immune factors for newborns. Roncada et al. (2012) reported that haptoglobins in whey are up-regulated in cows with subclinical and clinical mastitis. Fibrinogen is mainly known as a component of blood that infiltrates into the mammary gland and is increased in plasma after colostrum feeding (*Hernandez-Castellano et al., 2015a*). The fibrinogen alpha chain is the major precursor of blood clots (*Tamzali, Guelfi & Braun, 2001*) but also participates in inflammation, stimulating the adhesion, migration, chemotaxis, and phagocytosis of monocytes and macrophages at the point of infection (*Akassoglou & Strickland, 2002*). Similarly, mannose-binding lectin can alleviate the symptoms of *Mycoplasma pneumoniae* in sheep, enhance body resistance, and lessen inflammation (*Cosenza et al., 2012*). Growth-related protein, myosin light chain 6 (fragment), cyclin-g-associated kinase isoform x6, myostatin A, and connective tissue growth factor were also identified as DEPs, and all of these proteins showed decreases after 0 d. Dietary fat intake during infancy is very high, with approximately 50% of the total energy intake being acquired from milk lipids during the first month after birth (*Innis, 2007*). In the present study, lipoprotein lipase and apolipoprotein E expression levels also presented large differences, and fatty acid synthase (fragment) was identified as a DEP in the 3 d vs. 7 d group. In addition, several proteins involved in protein and nucleotide metabolism, such as histone H2B, are involved in the maintenance of the nucleosome structure of chromosomal fibers in eukaryotes (*Maruyama et al., 2006*); protein disulfide-isomerase functions in the regulation of protein misfolding; alpha-mannosidase is a key enzyme invovled in the modification of the eukaryotic protein N-glycan, which plays a decisive role in protein synthesis and conformational folding. The decline in the expression levels of these proteins may be correlated with mammary gland activity. In addition, the amounts of DEPs varied greatly in first 7 d after delivery, illustrated that adequate milk intake during this stage is critical to the development of the newborns.

Based on their GO functional annotations, the DEPs were classified in accordance with molecular function, cellular localization and biological pathways. In a previous study, 66% of the DEPs identified in the colostrum and mature milk whey of yak were found to be related to binding activity (*Yang et al., 2015*). Furthermore, researchers who analyzed ovine colostrum found that approximately 44% of the identified proteins were involved in catalytic activity and that 22.4% of the proteins were involved in binding activity (*Scumaci et al., 2015*). Our results were highly consistent with previous reports that catalytic activity and binding activity exist throughout the lactation period and account for the largest proportion of the total identified proteins. The molecular function category of the DEPs is most comprehensive in the 0 d vs. 3 d group, indicating that the whey proteins presented the most abundant function at 0 d after delivery. For example, antioxidant activity was only observed in the 0 d vs. 3 d group and was lost in the other groups, which also illustrated that colostrum is very important for providing protection against infections in newborns. Among the characterized processes, the proteins were classified into 17 categories, among which the metabolic process category was the largest and was detected in all time points. Another major process was the response to stimulus, suggesting that these proteins play a complementary role in other immunological and nonimmunological defense mechanisms in the gastrointestinal mucosa (*Ogundele, 2001*). In a total, the items of biological functions were least in the 56 d vs. 28 d group, which means that the milk whey proteins is at a stable state. Considering the argument of the early weaning time of sheep (*Ayadi et al., 2011; Ma et al., 2015*), the present study indicated that weaning at 28 d after delivery is feasible.

## Conclusions

This study explored the dynamic change tendency of Hu sheep milk whey proteins after delivery for the first time. Using iTRAQ proteomics technology combined with LC-MS/MS methods, a total of 310 whey proteins were identified and 121 DEPs were found. GO annotation analysis revealed that the biological functions of the proteins were most abundant in colostrum (4∼5 h after delivery) and lost in subsequent analysis gradually. The results from this research enriched the proteomics database of sheep milk whey, and are expected to provide a potential guidance for early weaning of lambs.

##  Supplemental Information

10.7717/peerj.10105/supp-1 Supplemental Information 1 The results of protein identification and relative expression abundance by matching to *ovis aries* proteomics database

10.7717/peerj.10105/supp-2 Supplemental Information 2 The results of peptide identification and relative expression abundance by matching to *ovis aries* proteomics database

10.7717/peerj.10105/supp-3 Supplemental Information 3 The results of protein identification and relative expression abundance by matching to *cetartiodactyla* proteomics database

10.7717/peerj.10105/supp-4 Supplemental Information 4 The results of peptide identification and relative expression abundance by matching to *cetartiodactyla* proteomics database

10.7717/peerj.10105/supp-5 Supplemental Information 5 Proteins identified at different lactation stages

10.7717/peerj.10105/supp-6 Supplemental Information 6 The 121 kinds of DEPs at different lactation stages

## Additional Information and Declarations

### Data Availability

The following information was supplied regarding data availability: The raw measurements are available in the Supplementary Files.
