# Supplementary material for: Exploration of ovine milk whey proteome during postnatal development using an iTRAQ approach
Source: PeerJ. 2020 Oct 8;8:e10105. doi: 10.7717/peerj.10105 (PMC7548079; doi:10.7717/peerj.10105)
Supplement: Supplemental Information 5 [file peerj-08-10105-s005.doc]

Supplementary Table 1 (Table S1) Proteins identified at different lactation stages.

| NO. | Accession NO. | Protein name | Species |
| --- | --- | --- | --- |
| 1 | G3LUQ4 | alpha s1 casein | *Ovis aries* |
| 2 | P04654 | alpha-s2-casein precursor | *Ovis aries* |
| 3* | W5Q6B8 | low quality protein: type ii cytoskeletal 6a isoform x1 | *Ovis aries* |
| 4* | W5PF70 | low quality protein: biorientation of chromosomes in cell division protein 1-like 1 isoform x1 | *Ovis aries* |
| 5 | W5NQP5 | superoxide dismutase [Cu-Zn] | *Ovis aries* |
| 6* | W5NXZ8 | g protein-regulated inducer of neurite outgrowth 1 | *Ovis aries* |
| 7 | E7BQS5 | alpha-s2-casein variant | *Ovis aries* |
| 8* | W5NUD7 | collectin-43 isoform x1 | *Ovis aries* |
| 9 | W5QB61 | peptidyl-prolyl cis-trans isomerase (fragment) | *Bos mutus* |
| 10* | W5QDQ1 | predicted: uncharacterized protein C1 or f94 homolog | *Ovis aries* |
| 11* | W5P0Q4 | haptoglobin isoform x1 | *Ovis aries* |
| 12 | Q9GK30 | parathyroid hormone-related protein (fragment) | *Ovis aries* |
| 13 | E7BQS2 | alpha-s2-casein variant | *Ovis aries* |
| 14 | A5YBU9 | complement factor B | *Ovis aries* |
| 15 | W5PJG0 | serum amyloid A protein | *Ovis aries* |
| 16 | Q9XSY9 | osteopontin | *Ovis aries* |
| 17* | W5Q160 | type i cytoskeletal 10 isoform x2 | *Ovis aries* |
| 18* | W5P3H7 | tpa: beta-ala-his dipeptidase | *Bos mutus* |
| 19* | W5Q5A6 | fibrinogen gamma chain isoform x2 | *Ovis aries* |
| 20* | W5NRG7 | inter-alpha-trypsin inhibitor heavy chain h4 isoform x1 | *Ovis aries* |
| 21 | W5PBZ7 | protein YIPF | *Ovis aries* |
| 22 | W5P7S6 | alpha-1-acid glycoprotein | *Ovis aries* |
| 23 | W5NQ46 | fibrinogen beta chain | *Ovis aries* |
| 24 | Q9MZY2 | airway lactoperoxidase | *Ovis aries* |
| 25* | W5PNC9 | melanoma 1 protein isoform x1 | *Ovis aries* |
| 26 | W5P408 | syndecan | *Ovis aries* |
| 27 | E7BQS3 | alpha-s2-casein | *Ovis aries* |
| 28* | W5Q124 | serpin a3-8 isoform x2 | *Ovis aries* |
| 29 | W5QGB5 | histone H4 | *Bubalus bubalis* |
| 33 | Q30DP5 | trefoil factor 3 (fragment) | *Bos mutus* |
| 34* | W5PID9 | complement component c9 | *Ovis aries* |
| 35 | W5Q5H8 | fibrinogen alpha chain | *Ovis aries* |
| 36* | W5Q0X5 | serpin a3-5 | *Ovis aries* |
| 37 | P42819 | serum amyloid A protein | *Ovis aries* |
| 38* | W5PG78 | potassium sodium hyperpolarization-activated cyclic nucleotide-gated channel 2 isoform | *Ovis aries* |
| 39 | C6ZP47 | I alpha globin | *Ovis aries* |
| 40* | W5PY10 | mucin-15 | *Ovis aries* |
| 41* | W5PW05 | malate mitochondrial isoform x1 | *Ovis aries* |
| 42 | W5QD52 | alpha-lactalbumin | *Capra hircus* |
| 43* | W5P737 | bpi fold-containing family b member 3 | *Ovis aries* |
| 44* | W5PRG8 | protein creg1 | *Ovis aries* |
| 45* | W5PYA9 | upstream-binding factor 1-like protein 1 | *Ovis aries* |
| 46* | W5NQQ0 | sushi repeat-containing protein srpx | *Capra hircus* |
| 47* | W5NXP6 | complement c3- partial | *Ovis aries* |
| 48 | Q29524 | lipoprotein lipase | *Ovis aries* |
| 49* | W5QA36 | lactase-like protein | *Ovis aries* |
| 50* | W5P6W8 | casein kinase i isoform beta | *Ovis aries* |
| 51 | P02075 | hemoglobin subunit beta | *Ovis aries* |
| 52 | W5NWF7 | elongation factor Tu (fragment) | *Ovis aries* |
| 53* | W5Q0L1 | elongation factor 1-gamma | *Ovis aries* |
| 52 | W5NWF7 | elongation factor Tu (fragment) | *Ovis aries* |
| 53* | W5Q0L1 | elongation factor 1-gamma | *Ovis aries* |
| 54* | W5PTS4 | ribonuclease 4 | *Ovis aries* |
| 55 | B5B304 | complement factor H (fragment) | *Ovis aries* |
| 56* | W5PMH6 | neutrophil gelatinase-associated lipocalin isoform x1 | *Ovis aries* |
| 57* | W5P1C2 | nucleobindin-2 isoform x1 | *Capra hircus* |
| 58* | W5P559 | odorant-binding protein 2b | *Ovis aries musimon* |
| 59* | W5P2K5 | type i cytoskeletal 18 | *Ovis aries musimon* |
| 60* | W5PE92 | low quality protein: granulins | *Ovis aries musimon* |
| 61* | W5PWS5 | dynein heavy chain axonemal isoform x1 | *Ovis aries* |
| 62* | W5PU70 | tpa: mucin-16 | *Bos taurus* |
| 63 | Q6IEB0 | putative ISG12(A) protein (fragment) | *Ovis aries* |
| 64* | W5Q6L8 | type i cytoskeletal 14 isoform x2 | *Ovis aries* |
| 65 | Q009B1 | ATP-binding cassette sub-family G member 2 | *Ovis aries* |
| 66* | W5Q611 | type ii cytoskeletal 1 isoform x2 | *Ovis aries musimon* |
| 67* | W5QH45 | kininogen-1 isoform x1 | *Ovis aries* |
| 68* | W5NSA6 | alpha-2-macroglobulin isoform x3 | *Ovis aries* |
| 69 | A6YRY8 | 40S ribosomal protein SA | *Ovis aries* |
| 70 | Q9XSQ2 | surfactant protein D (fragment) | *Bubalus bubalis* |
| 71 | W5PJR5 | serum amyloid A protein (fragment) | *Ovis aries* |
| 72* | W5PV41 | hhip-like protein 2 | *Pteropus alecto* |
| 73 | A2SW69 | annexin A2 | *Ovis aries* |
| 74 | P11839 | beta-casein | *Ovis aries* |
| 75* | W5PDM2 | fibroblast growth factor-binding protein 1 | *Ovis aries* |
| 76* | W5PSD8 | pancreatic secretory granule membrane major glycoprotein gp2 | *Ovis aries* |
| 77* | W5PCV9 | laminin subunit alpha-3 isoform x2 | *Ovis aries* |
| 78* | W5NU39 | fatty acid-binding protein | *Capra hircus* |
| 79* | W5PJA0 | protein isoform x4 | *Ovis aries musimon* |
| 80* | W5Q3W4 | ras-related protein rab-18 | *Bos taurus* |
| 81* | W5PHP7 | serpin a3-7-like | *Ovis aries* |
| 82* | W5PRX0 | pituitary tumor-transforming gene 1 protein-interacting protein | *Ovis aries* |
| 83* | W5PTZ8 | angiogenin-2 precursor | *Ovis aries* |
| 84* | W5NX51 | apolipoprotein a-i | *Ovis aries* |
| 85* | W5PF65 | low quality protein: serotransferrin isoform x1 | *Ovis aries* |
| 86* | W5NV32 | lipocalin-1-like isoform x5 | *Ovis aries musimon* |
| 87* | W5P6E0 | protein os-9 isoform x1 | *Ovis aries* |
| 88* | W5QFH5 | ras-related protein rab-1a | *Tupaia chinensis* |
| 89 | W5PUF2 | histone H2B | *Homo sapiens* |
| 90* | W5NZY6 | dnaj homolog subfamily b member 9 | *Ovis aries* |
| 91 | P02669 | kappa-casein | *Ovis aries* |
| 92* | W5P6F4 | complement c5 | *Ovis aries* |
| 93* | W5PP98 | glycine n-phenylacetyltransferase isoform x3 | *Ovis aries* |
| 94* | W5PCL4 | long chain fatty acid-ligase 1 isoform x1 | *Bison bison bison* |
| 95* | W5NZQ2 | cytosol aminopeptidase | *Ovis aries* |
| 96 | D2DRB7 | alpha-s1-casein variant | *Ovis aries* |
| 97 | Q9XSC0 | beta-lactoglobulin C (fragment) | *--* |
| 98* | W5NUH1 | cell death activator cide-a | *Ovis aries* |
| 99 | Q9XT27 | ceruloplasmin precursor | *Ovis aries* |
| 100 | Q9XSM0 | prostaglandin-H2 D-isomerase | *Ovis aries* |
| 101* | W5Q293 | dynein heavy chain axonemal isoform x2 | *Ovis aries* |
| 102 | W5Q9H2 | Protein disulfide-isomerase | *Ovis aries musimon* |
| 103 | P32262 | Antithrombin-III | *Ovis aries* |
| 104* | W5PZD0 | secretoglobin family 1d member-like | *Ovis aries* |
| 105 | W5PS45 | alpha-mannosidase (fragment) | *Ovis aries musimon* |
| 106* | W5NVC9 | ras-related c3 botulinum toxin substrate 1 | *Pan paniscus* |
| 107 | P09462 | Alpha-lactalbumin | *Ovis aries* |
| 108* | W5NSD5 | ras-related protein rap-1b | *Homo sapiens* |
| 109* | W5Q4T2 | protein kri1 homolog isoform x1 | *Ovis aries* |
| 110* | W5NPT7 | butyrophilin subfamily 1 member a1 | *Ovis aries* |
| 111* | W5Q4D0 | cyclin-g-associated kinase isoform x6 | *Ovis aries musimon* |
| 112* | W5NZ47 | retinol-binding protein 4 | *Bos mutus* |
| 113* | W5Q9A2 | zinc-alpha-2-glycoprotein isoform | *Ovis aries* |
| 114 | W5Q3X2 | peptidyl-prolyl cis-trans isomerase | *Pantholops hodgsonii* |
| 115* | W5NUX8 | complement c4 isoform x2 | *Capra hircus* |
| 116* | W5PD71 | c-reactive protein | *Ovis aries* |
| 117* | W5NVL1 | lysosomal-associated transmembrane protein 5 | *Ovis aries* |
| 118* | W5P1W2 | folate receptor gamma | *Ovis aries* |
| 119* | W5PE22 | rab gdp dissociation inhibitor beta | *Ovis aries* |
| 120 | A4ZVY8 | Beta-2-microglobulin | *Ovis aries* |
| 121* | W5Q9B6 | upf0464 protein like protein | *Bos mutus* |
| 122* | W5P7E7 | procollagen c-endopeptidase enhancer 2 | *Ovis aries* |
| 123 | Q7M371 | plasma proteinase inhibitor (fragment) | -- |
| 124* | W5Q3I2 | glycosylation-dependent cell adhesion molecule 1 | *Ovis aries* |
| 125 | W5PZI1 | clusterin | *Ovis aries* |
| 126* | W5Q629 | protein ddi1 homolog 1 | *Ovis aries* |
| 127* | W5NY46 | serum paraoxonase arylesterase 1 | *Ovis aries* |
| 128 | P50413 | thioredoxin | *Ovis aries* |
| 129* | W5PZJ1 | mammaglobin-a isoform x1 | *Ovis aries* |
| 130* | W5PH81 | complement component c7 isoform x1 | *Ovis aries* |
| 131 | P14639 | serum albumin | *Ovis aries* |
| 132* | W5Q268 | beta-2-glycoprotein 1 | *Ovis aries* |
| 133 | P00349 | 6-phosphogluconate dehydrogenase, decarboxylating | *Ochotona princeps* |
| 134* | W5QI04 | tropomyosin alpha-1 chain isoform x12 | *Ovis aries* |
| 135* | W5NS65 | histone -like | *Ovis aries* |
| 136* | W5PAJ2 | prosaposin isoform x6 | *Ovis aries* |
| 137 | Q5TIK9 | putative cyclophilin B (fragment) |  |
| 138 | D6PZY4 | factor H (fragment) | *Ovis aries* |
| 139 | F2YQ13 | gelsolin isoform b | *Ovis aries* |
| 140 | W5NW65 | tetraspanin | *Ovis aries* |
| 141 | W5P3X8 | kinesin-like protein | *Ovis aries* |
| 142* | W5QAB1 | hemopexin isoform x1 | *Ovis aries* |
| 143* | W5P0U4 | mucin-1 isoform x1 | *Ovis aries* |
| 144 | H9A6H7 | myostatin variant A (fragment) | *Ovis aries* |
| 145* | W5NSJ5 | serine threonine-protein kinase osr1 isoform x1 | *Ovis aries* |
| 146* | W5PFC9 | inhibitor of carbonic anhydrase-like isoform x3 | *Ovis aries musimon* |
| 147* | W5PSM5 | u6 snrna-associated sm-like protein lsm4 | *Ovis aries* |
| 148 | P53791 | sodium/glucose cotransporter 1 | *Ovis aries* |
| 149 | P68116 | fibrinogen beta chain (fragment) | *--* |
| 150 | I1WXR3 | alpha-1-antitrypsin transcript variant 1 | *Ovis aries* |
| 151 | W5QCD6 | Isocitrate dehydrogenase [NADP] | *Ovis aries* |
| 152* | W5QGX7 | eh domain-containing protein 4 | *Ovis aries* |
| 153* | W5NXP3 | serpin a3-6-like | *Ovis aries* |
| 154* | W5Q5J5 | t-cell antigen cd7 isoform x1 | *Ovis aries* |
| 155 | W5QH04 | amine oxidase [flavin-containing] | *Ovis aries* |
| 156* | W5NPK5 | complement c3 | *Ovis aries* |
| 157 | A6ZE99 | perilipin | *Ovis aries* |
| 158 | B0FZM4 | myosin light chain 6 (fragment) | *Rattus norvegicus* |
| 159* | W5NUV2 | adp-ribosylation factor 5 | *Tarsius syrichta* |
| 160 | W5QH64 | synaptosomal-associated protein | *Ovis aries* |
| 161* | W5Q2V0 | synaptobrevin homolog ykt6 | *Ovis aries* |
| 162 | S5FR89 | cathepsin B | *Ovis aries* |
| 163* | W5QH23 | mannan-binding lectin serine protease 1 isoform x1 | *Ovis aries musimon* |
| 164* | W5PCT4 | sodium-dependent phosphate transport protein 2b isoform x2 | *Ovis aries* |
| 165 | W5Q9P7 | cofilin-1 (fragment) | *Heterocephalus glaber* |
| 166* | W5P8F9 | short lung and nasal epithelium carcinoma-associated protein 2b-like | *Ovis aries* |
| 167* | W5Q302 | neutral alpha-glucosidase ab isoform x1 | *Ovis aries musimon* |
| 168* | W5Q5W1 | nucleotide exchange factor sil1 isoform x2 | *Ovis aries* |
| 169* | W5P0W4 | semaphorin-7a isoform partial | *Ovis aries* |
| 170* | W5PZS7 | alpha-1-antiproteinase isoform x1 | *Ovis aries* |
| 171* | W5PSA9 | vacuolar protein sorting-associated protein 13a | *Ovis aries* |
| 172* | W5PS94 | low quality protein: nucleobindin-1 isoform x1 | *Ovis aries* |
| 181* | W5Q1W8 | n-acetylglucosamine-1-phosphotransferase subunit gamma isoform x1 | *Ovis aries* |
| 182* | W5P101 | alpha-1b-glycoprotein isoform x2 | *Ovis aries musimon* |
| 183 | Q864L8 | peptidyl-prolyl cis-trans isomerase (fragment) | *Sus scrofa* |
| 184 | P04653 | alpha-S1-casein variant | *Ovis aries* |
| 185* | W5NWM2 | apolipoprotein a-iv | *Ovis aries* |
| 186 | P68214 | fibrinogen alpha chain (fragment) | -- |
| 187* | W5PTR5 | 78 kda glucose-regulated protein | *Ovis aries* |
| 188* | W5QCV4 | dehydrogenase reductase sdr family member 1 | *Ovis aries* |
| 189* | W5NQG4 | 60s ribosomal protein l4 | *Ovis aries musimon* |
| 190* | W5P3R3 | plasminogen isoform x1 | *Ovis aries* |
| 191* | W5PAY2 | protein wwc2 isoform x2 | *Ovis aries musimon* |
| 192* | W5PNP1 | lactadherin isoform x2 | *Ovis aries musimon* |
| 193* | W5NSH8 | epididymal secretory protein e1 | *Ovis aries* |
| 194* | W5PMT0 | xanthine dehydrogenase oxidase isoform x2 | *Ovis aries* |
| 195* | W5NTW3 | inter-alpha-trypsin inhibitor heavy chain h1 | *Ovis aries* |
| 196* | W5NZU3 | cadherin-1 isoform x1 | *Ovis aries* |
| 197 | W5PWE9 | serum albumin | *Ovis aries* |
| 198* | W5P979 | lymphocyte activation gene 3 protein isoform x2 | *Ovis aries* |
| 199 | B2MVW5 | RhoF | *Bos taurus* |
| 200 | Q30DR4 | macrophage migration inhibitory factor (fragment) | *Ovis aries* |
| 201 | W5Q695 | Transporter | *Ovis aries* |
| 202* | W5P812 | protein ambp | *Ovis aries* |
| 203 | Q6RUR7 | butyrophilin (fragment) | *Ovis aries* |
| 204* | W5PG95 | heat shock 70 kda protein 1b-like | *Ovis aries* |
| 205 | F5CC79 | beta-1,4-galactosyltransferase I | *Ovis aries* |
| 206* | W5P5I0 | complement factor i | *Ovis aries* |
| 220* | W5PJ97 | apolipoprotein a-ii | *Ovis aries* |
| 221* | W5NRI1 | complement c3 | *Ovis aries* |
| 222* | W5PSA3 | transcobalamin-1 isoform x5 | *Ovis aries* |
| 223* | W5P6B3 | phospholipid scramblase 1 | *Ovis aries* |
| 224 | C8BKD1 | prothrombin | *Ovis aries* |
| 225* | W5PTG9 | vitamin d-binding protein | *Ovis aries* |
| 226 | Q9MZS8 | cathepsin D (fragment) | *Ovis aries* |
| 227* | W5Q1W2 | syntenin-1 isoform x1 | *Ovis aries* |
| 228* | W5Q3T9 | programmed cell death 6-interacting protein | *Capra hircus* |
| 229 | C5IJ99 | RHOA | *Cricetulus griseus* |
| 230* | W5QDG7 | fibronectin isoform x1 | *Ovis aries* |
| 231 | O46544 | complement component C3 (fragment) | *Ovis aries* |
| 232* | W5PWT5 | cohesin subunit sa-2 isoform x2 | *Bos mutus* |
| 233* | W5PS44 | g-protein coupled receptor family c group 5 member b | *Ovis aries musimon* |
| 234* | W5PGE9 | immunoglobulin alpha heavy chain | *Ovis aries* |
| 235* | W5P256 | folate receptor alpha | *Ovis aries* |
| 236 | W5PFL8 | Tetraspanin (Fragment) | *Ovis aries* |
| 237* | W5NPN4 | heat shock cognate 71 kda protein | *Ovis aries* |
| 238* | W5P5T4 | complement c3 | *Ovis aries* |
| 239 | A0A059VBM8 | membrane cofactor protein | *Ovis aries musimon* |
| 240* | W5P3D6 | 40s ribosomal protein s14 | *Bubalus bubalis* |
| 241 | W5PZM9 | annexin | *Ovis aries musimon* |
| 242 | W5NVX6 | tetraspanin | *Ovis aries* |
| 243 | R4R2H5 | beta-casein (fragment) | *Ovis aries* |
| 244* | W5NTK7 | transmembrane protein c15orf27 homolog isoform x1 | *Ovis aries* |
| 245* | W5NU23 | plasma alpha-l-fucosidase | *Ovis aries* |
| 246 | W5PD15 | elongation factor 1-alpha | *Homo sapiens* |
| 247* | W5QG24 | palmitoyl-protein thioesterase 1 | *Ovis aries* |
| 248* | W5P9V5 | polymeric immunoglobulin receptor isoform x2 | *Ovis aries* |
| 249 | P29701 | alpha-2-HS-glycoprotein | *Ovis aries* |
| 250* | W5NUV1 | guanine nucleotide-binding protein g subunit beta-1 | *Pan paniscus* |
| 251 | P0CG55 | polyubiquitin-B | *Ovis aries* |
| 252* | W5P0U9 | angiopoietin-related protein 4 isoform x1 | *Ovis aries musimon* |
| 253* | W5P601 | erythrocyte band 7 integral membrane protein | *Ovis aries* |
| 254* | W5PPH6 | 40s ribosomal protein s3 | *Tupaia chinensis* |
| 255* | W5NWX6 | apolipoprotein c-iii | *Bos mutus* |
| 256* | W5PBM7 | retinoid-inducible serine carboxypeptidase | *Ovis aries* |
| 257 | C7DLN1 | fatty acid synthase (fragment) | *Ovis aries* |
| 258* | W5PQH0 | aminopeptidase n isoform x1 | *Ovis aries* |
| 259 | Q8HY29 | lipopolysaccharide receptor (fragment) | *Ovis aries* |
| 260 | K4P494 | cystatin C | *Ovis aries* |
| 261* | W5PAC0 | transmembrane protein 59 isoform x3 | *Ovis aries* |
| 262* | W5NT95 | renin receptor | *Ovis aries musimon* |
| 263 | Q7M2U8 | apolipoprotein E | *Ovis aries* |
| 264* | W5PDJ7 | 60s ribosomal protein l5 | *Panthera tigris altaica* |
| 265* | W5PUV9 | radixin isoform x2 | *Ovis aries* |
| 266* | W5QH54 | fetuin b | *Ovis aries* |
| 267 | H9CJU6 | 14-3-3 protein zeta/delta | *Ovis aries* |
| 268 | W5Q5G8 | Transketolase | *Ovis aries* |
| 269* | W5Q0Q1 | 14-3-3 protein theta | *Ovis aries* |
| 270* | W5QAR8 | noelin-3 isoform x1 | *Ovis aries* |
| 271 | M4WED3 | cell division cycle 42 | *Ovis aries* |
| 272* | W5P448 | glyceraldehyde-3-phosphate testis-specific isoform x1 | *Ovis aries* |
| 273* | W5PRJ4 | transitional endoplasmic reticulum atpase isoform x3 | *Ovis aries* |
| 274* | W5NXW9 | s25705ig mu chain | *Ovis aries* |
| 275 | W5PMM7 | protein disulfide-isomerase (fragment) | *Ovis aries* |
| 276* | W5PPQ8 | immunoglobulin j chain isoform x1 | *Ovis aries* |
| 277* | W5PHW0 | heat shock protein hsp 90-beta | *Bos taurus* |
| 278 | W5QC34 | alpha-mannosidase | *Ovis aries* |
| 279 | G8FRI7 | superoxide dismutase [Cu-Zn] (fragment) | *Ovis aries* |
| 280* | W5NUE3 | peroxiredoxin 1 | *Ovis aries* |
| 281* | W5QGM9 | melanotransferrin isoform x1 | *Ovis aries* |
| 282* | W5PN88 | elongation factor 2 | *Bos mutus* |
| 283* | W5NX95 | Ig lambda chain v-ii region bur-like isoform x2 | *Bos taurus* |
| 284 | A2P2I3 | VH region (Fragment) | *Ovis aries* |
| 285* | W5QI15 | immunoglobulin kappa-1 light chain variable region | *Ovis aries* |
| 286 | P60713 | actin, cytoplasmic 1 | *Camelus dromedarius* |
| 287 | W5Q927 | CD59 glycoprotein | *Ovis aries* |
| 288* | W5P8R7 | low quality protein: c-binding protein isoform x1 | *Ovis aries* |
| 289* | W5PM94 | acetyl-coenzyme a cytoplasmic isoform x2 | *Ovis aries* |
| 290 | W5PLB7 | peptidoglycan-recognition protein | *Ovis aries* |
| 291* | W5PHP6 | 60s ribosomal protein l27 | *Ovis aries* |
| 292 | P12303 | transthyretin | *Ovis aries* |
| 293* | W5P0V6 | saccharopine dehydrogenase-like oxidoreductase | *Ovis aries musimon* |
| 294* | W5QHZ8 | immunoglobulin kappa-4 light chain variable region | *Ovis aries* |
| 295 | W5NTD9 | chitinase-3-like protein 1 | *Ovis aries musimon* |
| 296* | W5QAL0 | glucosidase 2 subunit beta isoform x3 | *Ovis aries musimon* |
| 297* | W5P673 | pyruvate dehydrogenase phosphatase regulatory mitochondrial | *Ovis aries* |
| 298* | W5PK06 | low affinity immunoglobulin gamma fc region receptor ii-like isoform x3 | *Ovis aries* |
| 299* | W5PSP9 | immunoglobulin lambda-2c light chain variable region | *Pantholops hodgsonii* |
| 300* | W5PH95 | immunoglobulin heavy chain constant region of tetrameric 1a membrane form | *Vicugna pacos* |
| 301* | W5Q3H4 | ribosomal protein s2 | *Rattus norvegicus* |
| 302* | W5NV14 | immunoglobulin v lambda chain | *Ovis aries musimon* |
| 303* | W5PSQ7 | immunoglobulin lambda light chain f7-299 | *Bos taurus* |
| 304* | W5Q524 | poly polymerase alpha isoform x1 | *Ovis aries* |
| 305 | B3F206 | cryptochrome 1 | *Ovis aries* |
| 306* | W5PFM6 | iq domain-containing protein d | *Ovis aries* |
| 307* | W5PGT9 | immunoglobulin epsilon- partial | *Ovis aries* |
| 308* | W5NUN9 | mortality factor 4 like 1 | *Sus scrofa* |
| 309* | W5QHZ5 | Ig k protein | *Bos mutus* |
| 310* | W5PXV3 | connective tissue growth factor | *Pantholops hodgsonii* |

The absence of * indicates that the data was searched from Uniprot database, the addition of * indicates that the data was searched from NCBI database.
