# Supplementary material for: Exploration of ovine milk whey proteome during postnatal development using an iTRAQ approach
Source: PeerJ. 2020 Oct 8;8:e10105. doi: 10.7717/peerj.10105 (PMC7548079; doi:10.7717/peerj.10105)
Supplement: Supplemental Information 6 [file peerj-08-10105-s006.doc]

Supplementary Table 2 (Table S2) The 121 kinds of DEPs at different lactation stages.

| NO. | Accession NO. | Protein name |
| --- | --- | --- |
| 1 | G3LUQ4 | alpha s1 casein |
| 2 | P04654 | alpha-s2-casein precursor |
| 3* | W5Q6B8 | low quality protein: type ii cytoskeletal 6a isoform x1 |
| 4* | W5PF70 | low quality protein: biorientation of chromosomes in cell division protein 1-like 1 isoform x1 |
| 5 | W5NQP5 | superoxide dismutase [Cu-Zn] |
| 6* | W5NXZ8 | g protein- regulated inducer of neurite outgrowth 1 |
| 7 | E7BQS5 | alpha-s2-casein variant |
| 8* | W5NUD7 | collectin43 isoform x1 |
| 9 | W5QB61 | peptidyl-prolyl cis-trans isomerase |
| 10* | W5QDQ1 | predicted: uncharacterized protein C1 or f94 homolog |
| 11* | W5P0Q4 | haptoglobin isoform x1 |
| 12 | Q9GK30 | parathyroid hormone-related protein (fragment) |
| 13 | E7BQS2 | alpha-s2-casein variant |
| 19 | W5P7S6 | alpha-1-acid glycoprotein |
| 20 | W5NQ46 | fibrinogen beta chain |
| 21 | Q9MZY2 | airway lactoperoxidase |
| 22* | W5PNC9 | melanoma 1 protein isoform x1 |
| 23* | W5PY10 | mucin-15 |
| 24* | W5PW05 | malate mitochondrial isoform x1 |
| 25 | W5QD52 | alpha-lactalbumin |
| 26* | W5P737 | bpi fold-containing family b member 3 |
| 27* | W5NQQ0 | sushi repeat-containing protein srpx |
| 28 | Q29524 | lipoprotein lipase |
| 29* | W5QA36 | lactase-like protein |
| 30* | W5P6W8 | casein kinase i isoform beta |
| 31* | P02075 | hemoglobin subunit beta |
| 32 | W5NWF7 | elongation Tu |
| 33* | W5PTS4 | ribonuclease 4 |
| 34* | W5PMH6 | neutrophil gelatinase-associated lipocalin x1 |
| 35* | W5P559 | odorant-binding protein 2b |
| 36* | W5P2K5 | type i cytoskeletal 18 |
| 38* | W5PU70 | tpa: mucin-16 |
| 39* | Q6IEB0 | putative ISG12(A) protein (fragment) |
| 40* | W5Q6L8 | type i cytoskeletal 14 isoform x2 |
| 41* | W5Q611 | type ii cytoskeletal 1 isoform x2 |
| 42 | W5PJR5 | serum amyloid A protein (fragment) |
| 43* | P11839 | beta-casein |
| 44* | W5PDM2 | fibroblast growth factor-binding protein 1 |
| 45* | W5PJA0 | protein isoform x4 |
| 46* | W5Q3W4 | ras-related protein rab-18 |
| 47* | W5PTZ8 | angiogenin-2 precursor |
| 48* | W5QFH5 | ras-related protein rab-1a |
| 49* | W5PUF2 | histone H2B |
| 50* | P02669 | kappa-casein |
| 51 | D2DRB7 | alpha-s1-casein variant |
| 52 | Q9XSC0 | beta-lactoglobulin |
| 53 | Q9XSM0 | prostaglandin-H2D-isomerase |
| 54* | W5PZD0 | secretoglobin family 1d member-like |
| 55* | W5Q4T2 | protein kri1 homolog isoform x1 |
| 56* | W5Q4D0 | cyclin-g-associated kinase isoform x6 |
| 57* | W5NZ47 | retinol-binding protein 4 |
| 58* | W5NVL1 | lysosomal-associated transmembrane protein 5 |
| 59 | Q7M371 | plasma proteinase inhibitor |
| 60* | W5Q3I2 | glycosylation-dependent cell adhesion molecule 1 |
| 61 | W5PZI1 | clusterin |
| 62* | W5Q629 | protein ddi1 homolog 1 |
| 63* | W5PZJ1 | mammaglobin-a isoform x1 |
| 64 | W5P3X8 | kinesin-like protein |
| 65 | H9A6H7 | myostatin A |
| 66* | W5NSJ5 | serine threonine- protein kinase osr1 isoform x1 |
| 67* | W5PSM5 | u6 snrna- associated sm-like protein lsm4 |
| 68 | P68116 | fibrinogen beta chain (fragment) |
| 69 | I1WXR3 | alpha-1-antitrypsin transcript variant 1 |
| 70 | W5QH04 | amine oxidase [flavin-containing] |
| 71 | B0FZM4 | myosin light chain 6 (fragment) |
| 72* | W5PZS7 | alpha-1-antiproteinase isoform x1 |
| 73* | W5PSW9 | 60s ribosomal protein l8 |
| 74 | P04653 | alpha-S1-casein |
| 75 | P68214 | fibrinogen alpha chain (fragment) |
| 76 | B0BL71 | mannose-binding lectin |
| 77 | W5QHS2 | matrix Gla protein |
| 78* | W5P6B3 | phospholipid scramblase 1 |
| 79* | W5P3D6 | 40s ribosomal protein s14 |
| 80 | W5PZM9 | annexin |
| 81 | R4R2H5 | beta-casein (fragment) |
| 82* | W5NTK7 | transmembrane protein c15orf27 homolog isoform x1 |
| 83 | W5PD15 | elongation factor 1-alpha |
| 84* | W5QG24 | palmitoyl-protein thioesterase 1 |
| 85* | W5PPH6 | 40s ribosomal protein s3 |
| 86* | W5PQH0 | aminopeptidase n isoform x1 |
| 87 | Q7M2U8 | apolipoprotein E |
| 88* | W5PDJ7 | 60s ribosomal protein l5 |
| 89 | H9CJU6 | 14-3-3 protein zeta/delta |
| 90* | W5Q0Q1 | 14-3-3 protein theta |
| 91* | W5QAR8 | noelin-3 isoform x1 |
| 92* | W5NXW9 | s25705ig mu chain |
| 93 | W5PMM7 | protein disulfide-isomerase |
| 94* | W5PHW0 | heat shock protein hsp 90-beta |
| 95 | W5QC34 | alpha-mannosidase |
| 96* | W5NUE3 | peroxiredoxin 1 |
| 97* | W5QGM9 | melanotransferrin isoform x1 |
| 98* | W5QI15 | immunoglobulin kappa-1 light chain variable region |
| 99 | W5Q927 | CD59 glycoprotein |
| 100* | W5P8R7 | low quality protein: c-binding protein isoform x1 |
| 101 | W5PLB7 | peptidoglycan-recognition protein |
| 102* | W5PHP6 | 60s ribosomal protein l27 |
| 103 | P12303 | transthyretin |
| 104* | W5P0V6 | saccharopine dehydrogenase-like oxidoreductase |
| 105* | W5QHZ8 | immunoglobulin kappa-4 light chain variable region |
| 106 | W5NTD9 | chitinase-3-like protein 1 |
| 107* | W5QAL0 | glucosidase 2 subunit beta x3 |
| 108* | W5P673 | pyruvate dehydrogenase phosphatase regulatory mitochondrial |
| 109* | W5PK06 | low affinity immunoglobulin gamma fc region receptor ii-like x3 |
| 110* | W5PSP9 | Ig lambda-2c light chain variable region |
| 111* | W5PH95 | immunoglobulin heavy chain constant region of tetrameric 1a membrane form |
| 112* | W5Q3H4 | ribosomal protein s2 |
| 113* | W5NV14 | immunoglobulin v lambda chain |
| 114* | W5PSQ7 | immunoglobulin lambda light chain f7-299 |
| 115* | W5Q524 | poly polymerase alpha isoform x1 |
| 116 | B3F206 | cryptochrome 1 |
| 117* | W5PFM6 | iq domain- containing protein d |
| 118* | W5PGT9 | immunoglobulin epsilon- partial |
| 119* | W5NUN9 | mortality factor 4 like 1 |
| 120* | W5QHZ5 | Ig k protein |
| 121* | W5PXV3 | connective tissue growth factor |

The absence of * indicates that the data was searched from Uniprot database, the addition of * indicates that the data was searched from NCBI database.
